# Efficacy of Albendazole-Pyrantel Pamoate Compared to Albendazole Alone for *Trichuris trichiura* Infection in Children: A Double Blind Randomised Controlled Trial

**DOI:** 10.21315/mjms2020.27.3.7

**Published:** 2020-06-30

**Authors:** Eva Jacomina Jemima Sapulete, I Made G de Dwi Lingga Utama, I Gusti Ngurah Sanjaya Putra, Dyah Kanya Wati, I Made Arimbawa, I Wayan Gustawan

**Affiliations:** Department of Child Health-Medical School of Udayana University and Sanglah Hospital Denpasar, Bali, Indonesia

**Keywords:** albendazole, pyrantel pamoate, Trichuris trichiura

## Abstract

**Background:**

Soil transmitted helminths (STH) are intestinal nematodes and constitute one of the most neglected tropical diseases to exist. *Objective*: This study determined the efficacy of albendazole-pyrantel pamoate compared to albendazole in 8–12 years old children with *Trichuris trichiura* infection.

**Methods:**

A randomised controlled trial was conducted between October 2017 and February 2018 on participants whose stool examinations confirmed the presence of *Trichuris trichiura* infection. The subjects were randomised into two groups. The statistical analysis used Chi-square test.

**Results:**

There were 392 of 600 children at five public elementary schools in Bangli and Bali and were infected with *Trichuris trichiura*. The cure rate of *Trichuris trichiura* infection seven days following treatment was lower with the combination of albendazole and pyrantel pamoate compared to that of albendazole. The egg reduction rate of *Trichuris trichiura* infection was lower with the combination of albendazole and pyrantel pamoate compared to albendazole.

**Conclusion:**

The study indicated that a combination of albendazole and pyrantel pamoate does not improve cure rate or egg reduction rate in 8–12 years old children with *Trichuris trichiura* infection.

## Introduction

Soil transmitted helminths (STH) infections are considered to be intestinal nematode and constitute one of the most important but neglected tropical diseases to exist. STH can cause significant morbidity, disability, physical and intellectual growth, development delays, and impairments (1). STH infections are associated with humans and may include *Ascaris lumbricoides, Trichuris trichiura* and hookworm (*Ancylostoma duodenale, Necator americanus)* which are reported to have infected more than a quarter of the world’s population. The World Health Organization (WHO) reported that more than two billion people have been infected with *Ascaris lumbricoides*, 750 million with *Trichuris trichiura* and another 900 million with hookworm (2, 3). –

In the past ten years, it has been reported that Bali is an area with a high incidence rate of STH infection (4, 5). The most common types of STH in children are *Ascaris lumbricoides* and *Trichuris trichiura*. The highest prevalence rate exists in the age group of 8–12 years old children who have been residing in areas with high rainfall, humidity and local community with low rates education and poor environmental hygiene (6).

The four types of drugs that the WHO is determined to control STH with include albendazole, mebendazole, levamisole and pyrantel pamoate. Both types of benzimidazole groups have been widely used for STH infection, but those often require a combination with other drugs (7). It was reported that *Trichuris trichiura* therapy when combined with monotherapy had a lower cure rate compared to *Ascaris lumbricoides* infection with the same severity of infection (8, 9).

A study conducted by Speich et al. (9) reported that the cure rate (CR) of *Trichuris trichiura* was significantly higher with oxantel pamoate combined with albendazole compared to mebendazole (31.2%, 11.8%). On the other hand, Speich et al. (10) reported a significantly higher CR in *Trichuris trichiura* when treated with oxantel pamoate-albendazole (68.5%) (10). The study used oxantel pamoate which was not readily available in Indonesia and was used as an analog of pyrantel pamoate. Pyrantel pamoate had a trichurisidal effect that WHO recommends *adminiaa xm* for steered pyrantel pamoat to *Trichuris trichiura* infection (11). This study indicated that the efficacy of albendazole and pyrantel pamoate was higher compared to just using albendazole in children with *Trichuris trichiura* infection.

## Methods

This study was a double blind randomised controlled trial that aimed to determine the efficacy of combining albendazole with pyrantel pamoate compared to just albendazole in 8–12 years old children with *Trichuris trichiura* infection. The efficacy was determined using CR and egg reduction rate (ERR). It was seen that the CR for infected individual changed to ‘not infected’ within seven days of the study. The ERR is the difference between the number of *Trichuris trichiura* in the baseline data and seven days of monitoring compared to baseline data in line with WHO criteria. It was deemed satisfactory when the ERR value was ≥ 50% and decreased accordingly when ERR was < 50%.

The study was conducted at five public elementary schools in Bangli, Bali from October 2017 to February 2018. Children who were 8–12 years old were invited to provide stool samples, and children who tested positive for *Trichuris trichiura* were considered eligible for inclusion in the trial. Children who had diarrhoea, combination of STH infection, were taking STH medication within the past two months, vomiting within one hour after admiration of the drug, and the usage of certain drugs (benzimidazole and tetrahydropyrimidine) related to an allergic history were excluded.

The sample estimation was calculated using a hypothesis formula for two proportions with confidence interval at 0.05 and power of 80% with a difference of expected cure proportion set at 0.2. Twenty-eight subjects (children) were needed for each group. Based on a study by Adegnika et al. (12), the results showed that the effected proportion was 0.83 (P2) with the expected cure proportion was 0.2 based on a parasitology opinion, and therefore, P1 was set at 1.03.

The children were randomly assigned with the use of block size of two to receive one of two treatments. Group A was treated with combination of 400 mg albendazole and pyrantel pamoate 10 mg/kg body weight as a single dose for three consecutive days, while group B was treated with 400 mg albendazole in single doses for three consecutive days. The drugs were packed with same capsule cover. The medications were taken in the morning by the independent operator and supervised by the investigator. Subject and investigator were blinded to the treatment. There were independent teams used for blinding and randomising the groups. There was no industry involvement.

The researchers explained the purpose and procedures of the study including potential benefits and risks to parents or guardians of the children. The subjects who were willing to participate provided us with informed-consent forms signed by their parents. Subjects had collected stool samples. The stool was examined for worm eggs by using Kato-Katz’s methods. The stool was examined twice, once before the administration of anthelmintic agents and again seven days following treatment. Stool examinations were carried out at the Parasitology Department of Medical School, Udayana University by certified parasitology. Drug efficacy was assessed seven days after treatment. The statistical analysis was carried out using Chi-square tests with the significance level of *P* < 0.05. Finally, the collected data were analysed by SPSS software version 24.

## Results

**Figure 1:**
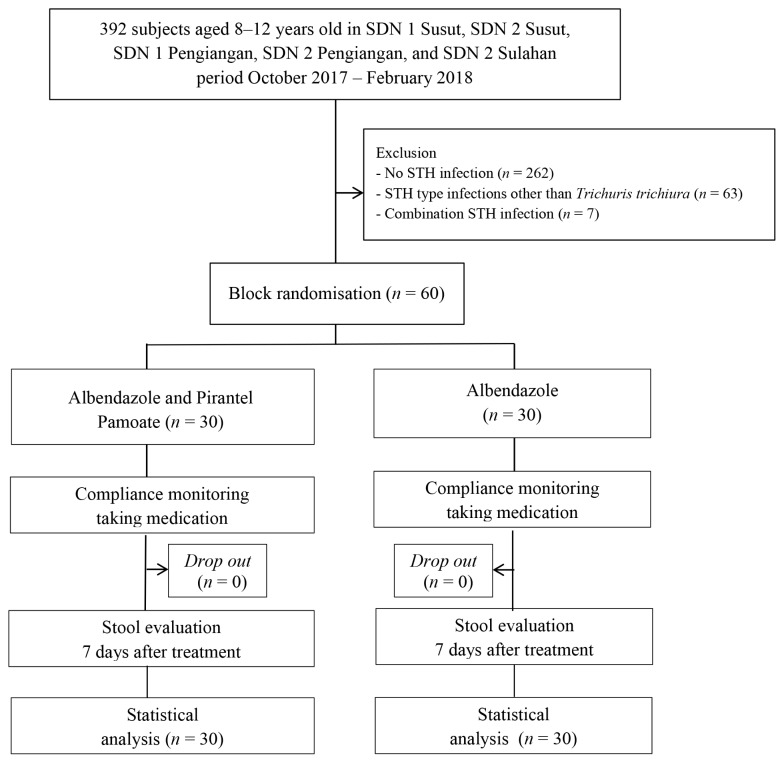
Study enrollment, randomisation and follow-up

There were 392 children who were aged 8–12 years old and were attending five public elementary schools in Bangli, Bali. Sixty subjects were infected with *Ascaris lumbricoides*, 60 were infected with *Trichuris trichiura* infection and three subjects were infected with *Enterobius vermicuaris*. The sixty subjects were analysed if they met the inclusion and exclusion criteria in the period of October 2017 to February 2018. Block randomisation was performed on 60 subjects. Each group consisted of 30 subjects. The study enrollment, randomisation and follow-up procedures are shown in Figure 1 below.

The characteristics of subject at baseline are shown in Table 1.

**Figure 2:**
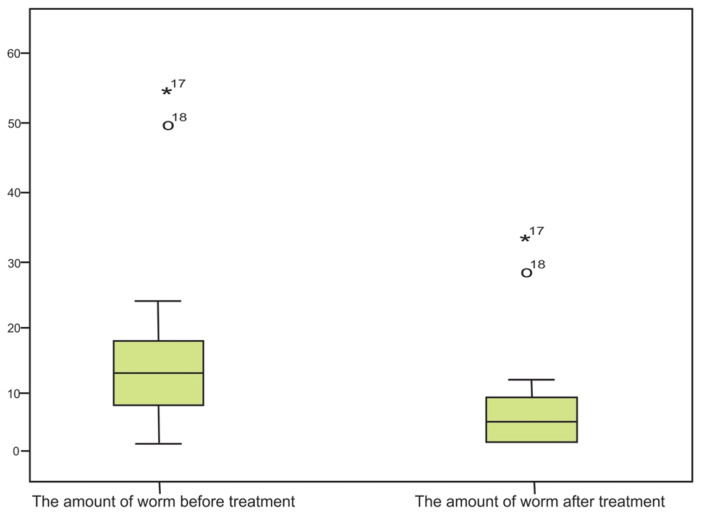
Box plot analysed egg worm count before and after treatment on albendazole-pyrantel pamoate group

**Figure 3:**
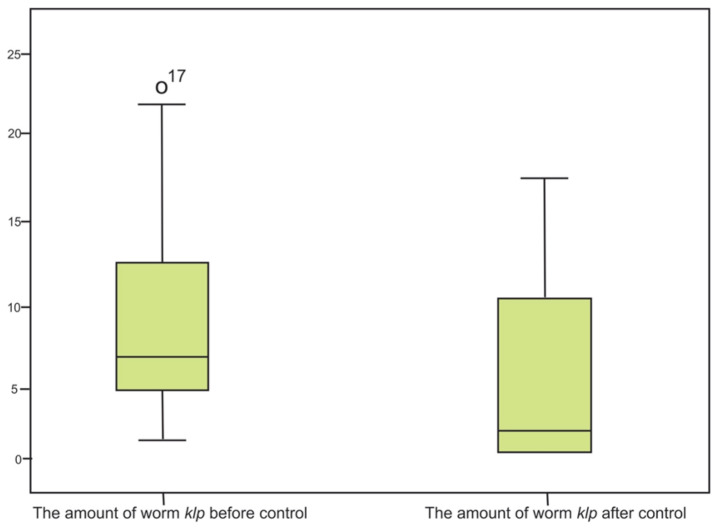
Box plot analysed egg worm count before and after treatment on albendazole group

The characteristics of subjects in the study included the following: 55% male and 45% female, nutritional status was 51.7% well nourished and 48.3% undernourished. All subjects came up as normal in the anthropometric status. The median (minimum/minimum-maximum/maximum) egg count before and after treatment in the albendazole-pyrantel pamoate group were (minimum 1, maximum 54, IQR 8.75) and three (minimum 0, maximum 14, IQR 8). Median (minimum/minimum-maximum/maximum) egg count before and after treatment in the albendazole group were 10 (minimum 4, maximum 24) and two (minimum 0, maximum 14). The analysed box plots are shown in Figures 2 and 3 below.

The CR and ERR among 60 subjects with *Trichuris trichiura* infection are shown in Tables 2 and 3. The CR of *Trichuris trichiura* infection was lower with the combination of albendazole-pyrantel pamoate compared to just albendazole (40% versus 60%, *P =* 0.19). The ERR of *Trichuris trichiura* infection was lower with albendazole-pyrantel pamoate than with albendazole (44.7% versus 55.3%, *P =* 0.28).

## Discussion

*Trichuris trichiura* infection is a chronic infection that can sometimes be asymptomatic but could lead to long-term effects that require appropriate treatment to prevent the impact of long-term effects. Mass treatment by WHO called prevention chemotherapy is still posing a challenge in eliminating STH infections in individuals. Treatment for *Trichuris trichiura* infection using two doses of albendazole was not reported to be optimal in the elimination of *Trichuris trichiura* infection.

It was found that albendazole when combined with pyrantel pamoate did not improve the CR and ERR as compared to administration of albendazole alone. This was different from the study done by Speich et al. (9) which used oxantel pamoate-albendazole. It was reported that some factors caused lower efficacy such as diagnostic methods, host and parasite characteristics and infection severity at baseline (13). This study used the Kato-Katz method for diagnostic evaluation. The limitation of Kato-Katz included lower sensitivity (88%) compared to FLOTAC methods (95%) (14). Sensitivity of diagnostic methods correlate to infection severity at baseline and Kato-Katz had low sensitivity in diagnosing whether the number of eggs were small (15). The infection severity was classified as a mild infection and the diagnostic method used was Kato-Katz as previously mentioned. These may have affected the outcome as sensitivity was mainly at baseline for diagnosis of mild infection.

Albendazole works by inhibiting the metabolism of parasite enzyme and it can also bind to β-tubulin and inhibits microtubule polymeration which has previously led to the destruction of cell structure and death of the parasite (16). The differences between pyrantel pamoate and oxantel pamoate lie in the cholinergic subtype. Pyrantel pamoate is an L-subtype and the N-subtype. The two subtypes differ in terms of the channel opening time, fastest N-subtype (0.6 ms) while the intermediate L-subtype (0.9 ms). This has affected both areas of pharmaco-dynamics and drug resistance (17). This study used a combination of albendazole and pyrantel pamoate in contrast to the Speich et al. (9) study that used albendazole-oxantel pamoate. The average difference between ion-channel opening time between pyrantel pamoate and oxantel pamoate may just be the cause of differing drug efficacy and it could be considered that pyrantel pamoate drug resistance to *Trichuris trichiura* infection still warrants further research.

It is recommended that evaluation after treatment should be no less than seven days and not more than 21 days. The WHO’s recommended time to assess efficacy is three weeks, but three weeks was considered to be too long and the ideal period was 10–14 days, because too long of a gap may effect immature stage maturation and not enough time would be left over to assess the efficacy of a good drug (18). Our study performed stool evaluation seven days after treatment. This affected the efficacy of the drug due to the presence of adult worms that do not die and were, consequently, able to produce new eggs.

The limitations of this study including certain diagnostic methods like the Kato-Katz laboratory technique which has low sensitivity in diagnosing *Trichuris trichiura*. Another limitation included conducting the stool evaluation seven days after treatment that could have affected the efficacy in our study.

## Conclusion

Albendazole combined with pyrantel pamoate did not improve the CR and ERR in this study compared to albendazole alone. This study highlighted that albendazole-pyrantel pamoate can both improve CR or ERR in 8–12 years old children with *Trichuris trichiura* infection. However, it can be concluded that albendazole alone is a sufficient treatment for mild trichurias among school-aged children.

## Figures and Tables

**Table 1:** Characteristic subject (*n* = 60)

No.	Characteristic	Group albendazole and pyrantel pamoate (*n* = 30)	Abendazole (*n* = 30)
1.	Age (year), mean ± SD	9.433 ± 1.250	9.333± 1.212
	Sex		
	Male, *n* (%)	14 (46.7%)	19 (63.3%)
	Female, *n* (%)	16 (53.3%)	11 (36.7%)
2.	Nutritional status		
	Good, *n* (%)	18 (60%)	13 (43.3%)
	Less, *n* (%)	12 (40%)	17 (56.7%)
3.	Toilet avaibility		
	Yes, *n* (%)	27 (90%)	27 (90%)
	No, *n* (%)	3 (10%)	3 (10%)
4.	Last medication		
	≤ 5 months, *n* (%)	22 (73.3%)	28 (93.3%)
	> 5 months, *n* (%)	8 (26.7%)	2 (6.7%)
5.	Antropometric status		
	Normal, *n* (%)	30 (100%)	30 (100%)
6.	Drug Compliance		
	Yes, *n* (%)	30 (100%)	30 (100%)
7.	Degree of infection		
	Mild, *n* (%)	30 (100%)	30 (100%)
	Moderate, *n* (%)	0 (0%)	0 (0%)
	Severe, *n* (%)	0 (0%)	0 (0%)

**Table 2:** Comparison of cure rate between groups

Group	Variable		df	*P-value*

	Cure	Not cure		
Albendazole and pirantel pamoate (*n* = 30)	10 (40%)	20 (57.1%)	1	0.19*
Albendazole (*n* = 30)	15 (60%)	15 (42.9%)		

Notes:

* Chi-square test; Chi-square value = 1.714

**Table 3:** Comparison of egg reduction rate between groups

Group	Variable		df	*P-value*

	Satisfactory	Decreases		
Albendazole and pirantel pamoate (*n* = 30)	17 (44.7%)	13 (59.1%)	1	0.28*
Albendazole (*n* = 30)	21 (55.3%)	9 (40.9%)		

Notes:

* Chi-square test; Chi-square value = 1.148
